# ENGAGING OLDER AND YOUNGER PEOPLE VIRTUALLY THROUGH THE ARTS

**DOI:** 10.1093/geroni/igae098.1509

**Published:** 2024-12-31

**Authors:** Krysta Peterson

**Affiliations:** Scripps Gerontology Center, Miami University, North Royalton, Ohio, United States

## Abstract

Following the COVID-19 Pandemic, the need for programming to foster connections and reduce loneliness for people of all ages increased. After the success of a three-year virtual OMA program, a new, video-chat application was developed to continue building intergenerational relationships virtually. ScrippsAVID (Arts-based, Virtual, Intergenerational, and Dementia-Friendly), pairs older and younger adults to discuss and co-create art, music, poetry, and share stories using over 75 art-based “prompts” designed to generate connections. Since its inception in 2023, ScrippsAVID has remained locally based with the majority of previous and current pairs comprised of Miami University students connecting with alumni. The program addresses older adults’ needs for social and creative engagement while providing younger adults opportunities to develop communication skills and build relationships with older adults, with and without dementia, outside of their families. Participants have commented on the value of their interactions with observations such as, “My AVID partner is one of the great joys in my life. Our time is so precious, and I am learning so much about life through her eyes. Thank you for this amazing opportunity. It is truly a blessing.” Overall, 132 older adults and 168 students have participated in ScrippsAVID with 90% of post-meeting satisfaction surveys reporting respondents being “Very Satisfied” (84%) or Satisfied (6%) with their meetup. This presentation will delve into the early ScrippsAVID implementation and outcome data to support further understanding of this innovative intergenerational program. We will also discuss potential future applications of ScrippsAVID for university-based intergenerational programming and community outreach.

